# Technical assistance: a practical account of the challenges in design and implementation

**DOI:** 10.12688/gatesopenres.13205.1

**Published:** 2020-12-03

**Authors:** Alexandra Nastase, Alok Rajan, Ben French, Debarshi Bhattacharya

**Affiliations:** 1 Oxford Policy Management, Oxford, UK; 2 Bill and Melinda Gates Foundation, New Delhi, India

**Keywords:** international development, technical assistance, capacity development, capacity substitution, capacity supplementation, policy options, state capability

## Abstract

Technical assistance is provided to strengthen state capability as part of international development programmes. This article presents the conceptual evolution of the technical assistance linked to capacity development, starting from a single unit of analysis, that of individual capacity to complex systems theories. It presents some of the frequent challenges in designing and implementing technical assistance, with a focus on the challenges common across government-funded and externally funded technical assistance, as well as the challenges unique to externally funded technical assistance. The article reviews the recent thinking on the practice in technical assistance from locally-driven technical assistance to positive deviance as a method to identify what works. It discusses change management as an adaptive and iterative process, and technical advisers acting as enablers of change rather than as change-makers and relying on adaptive and flexible approaches to programme management.

## Disclaimer

The views expressed in this article are those of the author(s). Publication in Gates Open Research does not imply endorsement by the Gates Foundation.

## Introduction

While there is no widely accepted definition of technical assistance, for this paper, we define it as knowledge-based technical assistance *contracted by or provided to* government to shape effective and inclusive policies, strengthening policy delivery, and building government capability.

**Figure 1.**
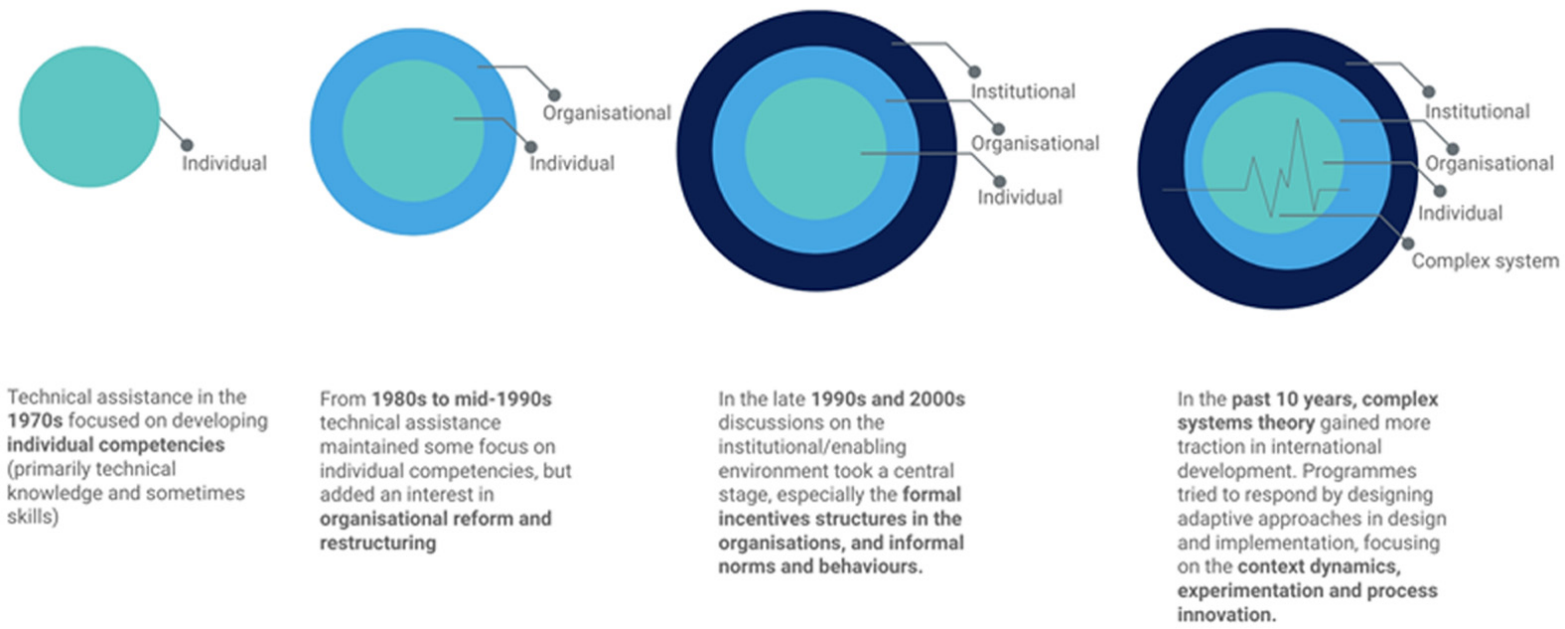
Evolution of technical assistance approaches linked to capacity development. Each circle represents one area of targeted for capacity development (individual, organisational and institutional capacity. In the last box, the lifeline icon pulse shows that the system has a life of its own and it is not only the sum of all the parts – the individual, organisational and institutional capacity.

In international development, technical assistance has been closely tied to capacity development. Figure 1 below summarises how the conceptualisation of capacity has been linked to the unit of analysis for technical assistance programmes. The World Bank has defined technical assistance as 'the transfer or adaptation of ideas, knowledge, practices, technologies, or skills to foster economic development for policy development, institutional development, capacity building, and project or programme support' ( World Bank, 1997). What is noticeable in the World Bank's definition is the implication of an external funder putting forward the idea of technical assistance to build or develop the ability of a government, to do something, as opposed to the government identifying a problem to solve and external partners contributing to the solution.

The first seeds of globally interconnected and geopolitical technical assistance were sown in the 1940s, with the establishment of the United Nations system and Harry Truman's ‘Point Four Program’ in 1949. Specifically, in the field of public health and academic capacity building, the Rockefeller Foundation (e.g. in its Mexico Hookworm eradication project; in its viral research in India, the Caribbean, Brazil, and Egypt; and through its Medical Sciences Division, which pioneered research in reproductive health etc.) established the paradigm of scientific neutrality in developing countries through the Foundation’s grants, projects, and fellows. In the 1950s and 1960s, technical assistance work was channelled towards filling experience gaps in developing country governments. This was achieved through the training of national staff and strengthening or restructuring of government institutions. In general, this approach to technical assistance relied on the use of technical experts from developed countries to work alongside and train recipient government staff. Over time, this led to core incumbent skills and functions that were supposed to be performed by the state officials being substituted by international technical advisers, and it did not achieve the expected long-term goal of developing effective national institutions for the government ( Cox & Norrington-Davies, 2019). While the currency, people, and language of internationally aided technical assistance has changed over the last few decades, with domestic expertise being called upon, the fundamental paradigm remains largely ‘external’.

From the 1980s onwards, thinking on technical assistance has shifted towards a broader view of capacity. Development organisations and governments have increasingly recognised, at least formally, that capacity development is about more than just building individual technical knowledge and skills. Building on the work of Douglas North, there has been a growing acceptance and Development of a framework that structures interactions into three categories: individuals, organisations, and institutions ( North, 1991) ^1^. This has shifted the focus on the enabling environment that provides the overall scope for change. The distinction between organisations and institutions is key to a realistic system-wide approach to capacity development that moves away from a singular focus on rules and policies, towards understanding informal barriers to change. This framework – institutions, organisations, and people (individuals) – remains highly relevant for understanding capacity and how organisations develop, and thus the role of technical assistance in enabling reform ( UNDP, 2009).

The most recent shift in how technical assistance is understood and conceptualised came in the 2000s. It built on complex systems theory, which assumes that ‘when smaller entities on their own jointly contribute to organised behaviours as a collective, [it results in] the whole being greater and more complex than the sum of the parts’ ( Paina & Peters, 2012). This is particularly relevant in the health sector, where many stakeholders interact to support the accessible delivery of care to a patient. In practice, many programmes ^2^ started to focus on asking process questions (‘how should we learn what works in the current situation?’), as well as trying to define best practice (‘what works?’) ( Husain, 2017). Experimentation, flexibility, and patience are core elements of programming in this ethos, and thus more flexible and adaptive models and research methods are required, to accommodate these key properties ( Smith & Hanson, 2011).

Additionally, a key condition is to reframe the intervention in complex systems as a learning process, rather than the implementation of blueprints and best practices. With this approach, the focus is on systems, and on how different elements interact dynamically, rather than on static units of analysis. As such, many programmes have moved the focus from a rigorous outputs-based programme design based on *what needs to be delivered* to a results-based programme design focused on *getting things done.*

## Key challenges for designing and delivering technical assistance

### Challenges common across government-funded versus externally funded technical assistance

A review of the literature on technical assistance highlights a series of challenges that are shared between government-funded technical assistance and externally funded, or third-party, technical assistance. Of these, attempts to transplant institutional models and best practice standards occur most often and have shown limited success in enabling governments to improve their functioning. It is increasingly well recognised that technical assistance often facilitates changes in the ‘form’ of institutions, without necessarily affecting their ‘function’. This issue has also been termed isomorphic mimicry ( Andrews *et al.*, 2012). In effect, while the assistance may help in the Development of technical systems and processes, it can be unsuccessful in changing the actual functions that an organisation performs, which is linked to the individual behaviours ( Williamson, 2015).

This challenge is particularly acute in environments where external financing of government functions is critical (e.g. when governments are dependent on development financing, or where state governments depend on significant federal transfers to finance their budgets). Repeated isomorphic mimicry projects, policies, and programmes lead to capability traps – a cycle whereby governments are constantly adopting reforms, partly to ensure ongoing flows of external financing and legitimacy, without any tangible improvements. These capability traps emerge when there are efforts to reproduce templated solutions that are considered ‘best practice’ through predetermined linear processes, or where there is an overbearing adherence to a rigid plan for implementation ( Andrews *et al.*, 2012). For instance, there are indications that fragile and conflict-affected states are quicker to adopt results-based financing in health as compare to more stable countries, given their dependence on external funding, but also weak governance arrangements (Bertone *et al.*, 2018)

Unclear expectations from capacity development initiatives exacerbate challenges related to isomorphic mimicry and capability traps. There are various reasons for this, starting with programme designs that include unrealistic theories of change and action, logic frameworks that focus on outputs and not outcomes (e.g. the number of people trained), and continuing with implementation challenges such as managing donor or government expectations with regard to the role of advisers. Frequently, technical advisers are expected to perform government functions outside the terms of reference, and sometimes substituting government functions even if the agreement was to supplement or build capacity. When this happens, some consultants may face the dilemma of responding to the request and thus build trust in the relationship with the government counterparts or push back and risk damaging the relationship. In practice, this is a difficult situation to manage and it takes commitment, and experience to navigate it in the benefit of the programme.

Patience is also a critical factor when it comes to capacity development, especially when there is external pressure on governments to deliver on targets and global agendas (including, for instance, the Millennium Development Goals, the Sustainable Development Goals etc.). Building capacity takes time: it is not risk-free and sometimes it may imply compromising the quality of results and the capacity to deliver quick wins.

Also, often sustained capacity in incumbent systems is built when the system discovers its own problems – both small and large, tests their own solutions and fail themselves – essentially transforming itself into a thinking, learning and hence, a responsive and resilient organisation. External technical assistance needs to act concurrently and patiently in breaking the inevitable falls and accelerate learning of important lessons, without prejudice or predisposition towards a templated ‘solution’.

These factors make it difficult – and sometimes even impossible – to agree on the result a technical assistance provider should seek to achieve. Should they build capacity, replace capacity and just deliver the function, or do something in the middle? Unfortunately, without clarity of the objective, technical assistance programmes oscillate between objectives: substituting capacity to produce short-term progress, but not addressing the underlying capability challenges, thus leaving the system to revert to ‘business as usual’ as soon as the technical assistance ends.

Regular changes in political priorities, or the transfer of key personnel, is often a significant barrier to lasting change in government capability. Large-scale, long-term technical assistance programmes typically do not have the same leadership at the end of the programme, or at crucial decision-making points in the middle of the programme, as they started with. Political and bureaucratic bipartisanship is often not a design feature of technical assistance programmes when they are launched. Frequent political, bureaucratic, and technocratic changes are also a defining characteristic of weak state capacity. The extreme alternative to frequently changing regimes and cadres in weak states, is an authoritarian rule, which is defined by its extractive nature, especially from external donors and aid-funded technical assistance programmes.

Inflexible theories of change and action of technical assistance programmes also hamper the holistic visioning of the pathway to long-term impact and goals. No sub-system (e.g. health, agriculture, sanitation etc.) operates in a silo of its own: they have interdependencies with other sub-systems. For example, in water resources management, there is an intricate nexus between how agriculture, energy, and ground water interact and impact each other. Not considering these interdependencies, and potential extraneous changes in the environment means that while much technical assistance has robust logframes and hypotheses, they ultimately have weak narratives on the pathways of systemic and long-term change. Also, the theories of change need to be anchored in the organisational and institutional context, which may also refer to understanding different streams of work including other ongoing initiatives of international partners, national authorities, non-state actors.

Finally, the projectized nature of technical assistance is a structural challenge. Although technical assistance engagements are signed off between the host government and donor agencies, the resources deployed on the ground – from technical experts at the highest level to the implementation support resources on the ground – typically are employed and deployed by local/international non-governmental organisations. These resources’ careers also typically move from one technical assistance project to another, funded by one agency or another. The tendency for people to seek to prolong their careers also plays out in how truly sustainable reforms eventually become (or not), as the same technical assistance resources are expected to prepare the system to wean itself of themselves, having built sufficient capacity effectively. In essence, the providers are expected to work themselves out of a job. Technical assistance is also projectised in nature due to the way public procurement rules are structured in government. Building genuine capacity through technical assistance can have ambiguous pathways of change that need to be built along the way. Most government procurement structures are not designed to support projects of this kind, but are rather focused on making procurement better defined, with clear targets and deliverables, and a strong focus on value for money.

### Challenges unique to externally funded technical assistance

Technical assistance programmes tend to focus on technical solutions to what are often social and political problems. In doing so, there is also a tendency to overlook rich local knowledge and fit-for-context solutions that can work well. The literature suggests that standard models for improving public sector management have little impact on the way bureaucracies or political systems work in low-income settings ( Cox & Norrington-Davies, 2019). An underlying assumption in traditional models of public sector reform is that development issues are technical problems and arise out of information asymmetry. Hence, given better information about government performance and citizens’ entitlements, actors will behave differently, and the supply of public goods will improve. With the limited success of large-scale external technical assistance, this assumption has been challenged, and the literature suggests that coordination and collective action challenges influence the behaviour of actors much more than information asymmetry does ( Booth, 2014).

Most development programmes often assume a one-way learning relationship, where the recipient is learning form the provider. In contrast, in practice, there is often a two-way exchange of ideas and ways of working. For example, donor support to anti-corruption reforms in Malawi failed due to the ‘cargo transfer’ to Malawi of anti-corruption laws and structures that did not take account of Malawi’s implementation capacity ( Cox & Norrington-Davies, 2019).

As problematic, these one-way learning relationships encourage silos in the provision of technical assistance programmes. For instance, in global health, many actors may be genuinely committed to address the issue of coordination, they face a series of challenges, such as proliferation of global health actors; problems of global leadership; divergent interests; problems of accountability; problems of power relations (Spicer *et al.*, 2020). This often leads to projects not being strategic, and limited coordination leads to overlaps between donors, and, in certain cases, contradictory or inconsistent reform advice ( Cox & Norrington-Davies, 2019). External technical assistance providers are bound by their individual contract and terms of reference with the funding institution and the host government. This formally shifts the onus for coordination between external parties largely onto the host government, which has weak capacity to begin with, perpetuating a vicious cycle dominated by an inter-partner dynamic where host governments take a backseat. Government ownership of critical reforms suffers in the process.

### Learning from international practice in providing technical assistance

The past 10 years in international development have been characterised by an increased interest in defining more politically astute programmes. At the foundation of this movement is practitioners’ understanding that the mixed results of Development, and particularly capacity development programmes, have less to do with a lack of knowledge or funding and more with the power structures that allow actors, groups, or collective movements to gain from existing movements and to resist change ( Leftwich & Sen, 2011). As a result, different approaches emerged, involving principles, methods, and tools to operationalise more adaptive, politically informed ways of thinking and delivering technical assistance. These approaches include the following.

• **Thinking and working politically** is based on three core principles: strong political analysis, insight and understanding; a detailed appreciation of and response to the local context; and flexibility and adaptability in programme design and implementation ( TWP CoP, 2013).

• **Development entrepreneurship** is a form of thinking and working politically that postulates that development entrepreneurs work within a wider process of coalition building, using iterative learning by doing and making small bets to find ways to introduce reforms ( Faustino & Booth, 2014).

• **Problem-driven iterative adaptation** rests on four principles: local solutions to local problems; pushing problem-driven positive deviance; trying, learning, iterating, and adapting; and scaling up through diffusion ( Andrews *et al.*, 2015)

• **Doing development differently** involves five potential starting points: swimming against the tide; working in and with government; feedback loops and data; organisational change; and diffusion ( Wild & Andrews, 2015)

• **Adaptive management** is an intentional approach to making decisions and adjustments in response to new information and changes in context ( USAID, 2016).

• **The World Bank’s Global Delivery Initiative** is a platform that aims to bring together knowledge of what works, and practitioners, to strengthen policy and programme delivery.

A few principles are common across these initiatives:

**Locally driven technical assistance focused on developing local capacity**. There is general acceptance that the only way technical assistance can create sustainable capacity is through ensuring that local problems are owned by and solved by local actors. Local actors are much more likely to have the motivation, credibility, knowledge, and networks to mobilise support, leverage relationships, and identify opportunities in politically astute ways, as compared to their external counterparts ( Andrews *et al.*, 2012). That said, mobilising local support is not a straightforward task, and therefore technical assistance providers need to ensure that the problems that technical assistance programmes seek to address have a high level of salience to local actors.

**Focus on addressing small problems.** Two aspects require further consideration: focusing on problems instead of solutions and focusing on small bets to learn from, iterate, and scale up. For a long time, scholars have discussed the need to shift from solution-focused development programming to a problem-driven approach. However, the practice has been slow to follow through on this. As we speak, best practice solutions are implemented around the world in response to COVID-19, with little recognition for the particularities of context and understanding of the enabling conditions required for success. The problem is aggravated when the solutions are large reform programmes that capture a large amount of public resources. By contrast, entrepreneurship thinking suggests that many small bets are better than one large bet. Small bets make failure less costly and thus increase the degree of learning and innovation, and eventually, the effectiveness of the solutions. 

**Diffusion, through positive deviance.** Part of the theory of change for some of the approaches mentioned above – although sometimes not explicitly mentioned – is positive deviance. This rests on the premise that the contexts in which development practitioners act in are complex, and for any given problem, someone in the community will have already identified a solution ( Green, 2016). Learning from how the solutions were possible in the first place, and supporting their diffusion within the system, is what makes a difference in Development. This is also the theory of change for some ‘pockets of effectiveness literature’ ( Hickey, 2019), although there is little evidence on their spill-over effects as regards improving performance outside the remits of the organisation these are part of ( Roll, 2014). Organisations are much more likely to pursue the path of gradual and incremental reform, through the resolution of specific problems, rather than reform by following a set blueprint.

**Change as an adaptive and iterative process**. There are at least two type of problems to be solved with technical assistance: logistical, with clear, proven ways of working; and wicked, involving system-wide approaches ( Andrews *et al.*, 2015). For the second category, there is a growing recognition that reform pathways are not linear, or even technical, but deeply rooted in the political and social context of recipient countries. This has led to finding ways of working adaptively and flexibly. The core of these new ways of working is defining portfolio approaches to solve problems, and deliberate ways of testing, learning, and experimenting while supporting change ( Cox & Norrington-Davies, 2019). The main assumption is that complex change programmes cannot rely on defining the entire journey to reform upfront, but rather require defining the destination and being flexible about the entry points to get to that destination.

Adaptive management is an umbrella concept for the adaptive approach to programme design and implementation, which focuses on learning and adaption in order to get things done. Its premise is that the change we are usually seeking in Development is difficult and sometimes unpredictable, and in these conditions, the decision-making process cannot be linear, it must be iterative. Starting a journey with a GPS navigator that does not update itself based on the encountered road conditions will not take you to your destination.

More specifically, an adaptive management framework allows the programme implementers to draw on the relevant type of technical assistance at key points to push for change. In literature, this is usually referred to as having a portfolio approach. The government has a menu of options to choose from and they may build towards the final end-goal by experimenting with what works. Many development projects work on a model whereby they use in-sourcing to advance on an important task that they do not have capacity for but that they need delivered quickly (the demand-driven capacity substitution), and then they use specific technical inputs to understand a public sector delivery problem (capacity supplementation), which will be solved by the inter-departmental working groups facilitated by an external adviser (capacity development).

**Technical assistance providers as facilitators rather than doers.** At the heart of the emerging lessons is a growing recognition that if the goal is capacity development, the role of the technical assistance provider needs to change from that of an external resource brought in to do work on behalf of the government, towards that of a facilitator of change ( Andrews *et al.*, 2012).The literature strongly supports this new approach, although evidence of its effectiveness is still emerging ( Cox & Norrington-Davies, 2019). An important prerequisite for this change to robustly establish itself as a paradigm is a deep sense of understanding and ownership of the problem by the incumbent government. This can be hastened and deepened when external technical assistance is intentionally indigenised, both in form and function. It takes a lot for a weak recipient of external technical assistance to not succumb to the purported urgency and professionalism of external technical assistance.

Experience shows that poorly delivered technical assistance can displace and even erode national capacities ( Cox & Norrington-Davies, 2019). In addition, unintended capacity substitution can occur where poor working relationships exist between external experts and government staff. These relationships can be affected by disparities in salaries, equipment, and other softer elements, which leads to low morale within government counterparts ( Le *et al.*, 2016). The perception that external advisers are superior personnel can disrupt hierarchies within government organisations, leading to a less than conducive work environment ( Hadebe, 2016). This is exacerbated in places where the legislature (i.e. politicians) cannot or does not fully rely on the executive to implement most of its policies. This characteristic also exposes fractured accountability between politicians and the executive, and between the executive and consumers of public services.

**The relationship between the government counterparts and external advisers is a crucial dynamic for the type of technical assistance being delivered.** From our experience, the staffing of technical assistance programmes may influence the shape the assistance takes. For instance, junior consultants or external staff who are paid significantly lower than government counterparts may end up doing capacity substitution work as they are perceived to ‘assist’ the government. At the opposite end, the senior consultants who may be compensated significantly above the government salaries may be accepted as partners or change facilitators. Still, there will also invariably be an expectation that they will intermediate the relationship with the superiors because communication within government systems is aligned with power and hierarchical rules. Ideally, this would be addressed by properly managing expectations upfront among all relevant parties, starting with the political leadership, the bureaucracy, the donors, and the external consultants.

## Application of technical assistance to health system strengthening

Technical assistance in public health over the years has tended to focus on vertical disease programmes, or health system strengthening. In the initial years, the focus was squarely on the former, but there was soon recognition that support to broader health system pillars is essential to ensure better delivery of vertical programmes ( Chee *et al.*, 2013). This led to many donors pivoting towards health system strengthening projects, all with varying interpretations of what health system strengthening mean, and varying areas of focus. Initial conceptualisations of a health system used a ‘building blocks’ approach – dividing a health system into six parts: health workforce; health information systems; supplies and infrastructure; finance; governance and leadership; and service delivery. Health system strengthening approaches have often focused on individual building blocks.

In recent years, this mechanistic, linear view of a health system – and its implications for how to go about health system strengthening has been challenged ( Chee *et al.*, 2013). There is a recognition that whilst the building blocks – which are seen as the ‘hardware’ of a system – are of course important, so are the people in the system – the ‘software’ ( Sheikh *et al.*, 2011). The extent to which hardware components translate into effective, quality service delivery depends on the behaviours and interactions of the people in a system. The behaviours that are governed both by ‘tangible software’ (their capacities and the formal processes that a system mandates) and ‘intangible software’ (the norms, values, incentives, relationships and culture that influence behaviour in practice).

Software considerations are rooted within the broader social and political context of a health system. Hardware and software considerations dynamically interact: for example, the availability of resources affects provider motivations, and it is this dynamic interaction that determines the outcomes produced by a health system. Thus, health systems are increasingly seen as ‘complex adaptive systems’ (or ‘systems of systems’, given the number of component parts of a health system, all of which interact in complex ways). A field of applied research focusing on these issues – known as Health Services Research and Policy – has become increasingly prominent.

The implication of these conceptual advances in the health systems literature is that strengthening a health system can no longer be viewed as a technical, linear problem that can be solved through rational planning. Desired outcomes (whether resilience, performance, responsiveness, or respectfulness) are not things that can be simply achieved through strengthening inputs, particularly individual blocks. Rather, there is a need to create an enabling environment so that these outcomes emerge from the dynamic interactions between hardware, software, and different levels of the health system.

The health systems literature has made significant conceptual and empirical progress on identifying the components of the enabling environment required to achieve improved outcomes – particularly the importance of trust, pro-social values, teamwork, and distributed leadership. These tend to be common across different types of desired outcomes studied.

However, there has been significantly less progress made in identifying interventions that can be successful in bringing about this enabling environment. Some of the interventions identified that have an emergent evidence base include training and coaching on supportive supervision; coaching and mentoring on transformational leadership; the creation of peer-networks; and social accountability. However, these only have limited traction in large health system strengthening projects – partially because they challenge the predominant approaches used in large technical assistance programmes. There is no conclusive evidence on whether externally funded and provided technical assistance programmes are even the right vehicles for delivering systemic change in the ‘software’ of health systems, despite them having successfully diagnosed a problem.

Another interesting distinction is made in the literature between health system strengthening and health systems support. Chee defines health system strengthening as ‘about permanently making the systems function better, not just filling gaps or supporting the systems to produce better short term outcomes’ ( Chee *et al.*, 2013). In certain cases, both health system strengthening, and health system support can exist in the same programme, especially ones that cover multiple decentralised levels of the public health system. That said, it is not uncommon for such programmes to become unwieldy and there is a risk that the technical assistance at the more central levels becomes focused on enabling support at the service delivery levels and that the support at the service delivery level becomes substitutional.

While the adage ‘What gets measured gets done’ is extremely appropriate in evaluating health system strengthening efforts, its unfortunate corollary is also often true: ‘Only what can be measured gets done’. Evaluation and measurement has not kept pace with sophisticated/nuanced approaches to measuring changes in the capacity and/or ‘software’ of health systems, development partner-funded projects tend to continue to measure inputs and classical ‘coverage’ indicators, which perpetuates continued emphasis on doing things or getting things done, instead of on developing capacity.

## Learning sites

The principles of health system strengthening highlighted above have been applied in different country contexts in recent times. Some of the work done in South Africa and Kenya by Lucy Gilson finds that governments and donors must strengthen leadership across systems, and this can be achieved through new forms of collaboration. Technical assistance between researchers and health managers may build resilience, and this is achieved through researchers becoming embedded within the social networks surrounding and supporting health systems ( Gilson *et al.*, 2017).

As part of the research, three learning sites were embedded in two different national contexts: in Kilifi County in Kenya, and in two health districts located in different provinces of South Africa. The learning sites approach has put into practice many of the principles of emerging technical assistance models that were discussed earlier in this note:

**1.   Local actors** – Learning sites are based on collaboration between researchers and health managers: research is conducted with practitioners rather than about their practice. Research-practitioner partnerships are formed between groups rather than individuals; they are multi-layered, dynamic, and interdependent ( RESYST, 2016).

**2.   Iterative** – Various approaches to research are focused on an evolving set of issues, including research that does not start with a predefined question. The overall process of enquiry is emergent within a broad framework, and with a commitment to joint ownership and co-production of knowledge ( Gilson *et al.*, 2017).

**3.   Locally nominated problems** – Researchers are embedded in the health system; through research activities they come to understand the daily routines and challenges faced by health managers, and become part of the social network of learning site colleagues ( RESYST, 2016).

Gilson’s research concludes that health system strengthening must pay closer attention to the software of health systems. Building resilience is not merely about equipping the system with more hardware and technology, it is also about developing people’s agency. As the author states: ‘resilience specifically is nurtured by developing the internal organisational capacities needed to adjust to and learn from routine challenges and preserve or even improve health system functioning. The forms of health system strengthening must guard against undermining these capacities’ ( Gilson *et al.*, 2017). The learning site experience suggests that the long-term process of health system change requires being embedded in the context, to understand complex and long-term processes and challenges, but also to maintain the balance between genuine strengthening and cosmetic support.

## Reflections for designing effective technical assistance

This article aimed to present an account of the theory and practice of technical assistance, insisting on some of the challenges in implementation and the sometimes-difficult trade-offs governments and donor need to consider when designing technical assistance. To support the decision-making process, we suggest below a series of questions that can guide difficult conversation, in which technical assistance is a policy options for governments to advance the country’s development objectives.

- How can technical assistance contribute to the country’s development objectives?
- What are the problems to be addressed? Or Is this an intervention focused on implementing a solution? Have the government and the development partner considered the implications of a problem-driven and a demand-driven approach?
- What role will the government have during the implementation of technical assistance (managing a contract, driving a process etc.)? What support do they require as part of technical assistance programmes?
- What role is the development partner expected to play?
- Does the envisaged work account for a complex system, including existing interdependencies or is it considering only separate parts of the system – the individuals, organisational setup, legal framework etc.?
- How is the work planned and how will risks be managed – will the implementers work within a framework that is focused on results and thus allow for course correction when the conditions change?
- How is learning integrated in the programme – including government learning and learning from what works in reform programmes in that particular contexts and the enabling conditions?
- How will the programme balance short-term inputs with long-term sustainable change?
- What are the conditions and resources available for implementing partners to deliver on the agreed principles above? (experience with managing adaptive frameworks, managing risks, deploying various types of resources, staffing qualifications and balance of local and international etc.)

## Data availability

No data are associated with this article.

## Footnotes

^1^ This type of three-level framework is not uncommon. For similar distinctions see North (1991), OECD (2008), UNDP (2009).

^2^ Development partners have embraced this ethos of working to various degrees, based on criteria such as their relationship with the government, how they have traditionally worked with their counterparts, their appetite for risk, and their patience as regards waiting for change.
