# Technical assistance: a practical account of the challenges in design and implementation

**DOI:** 10.12688/gatesopenres.13205.2

**Published:** 2021-11-19

**Authors:** Alexandra Nastase, Alok Rajan, Ben French, Debarshi Bhattacharya

**Affiliations:** 1 Oxford Policy Management, Oxford, UK; 2 Bill and Melinda Gates Foundation, New Delhi, India

**Keywords:** international development, technical assistance, capacity development, capacity substitution, capacity supplementation, policy options, state capability

## Abstract

Technical assistance is provided to country governments as part of international development programmes to support policymaking or strengthen state capability. This article presents the conceptual evolution of ‘technical assistance’ linked to capacity development, starting with programmes aiming exclusively to enhance individual capacity in the 1950s to 1970s and progressing to complex systems approaches in the past ten years. It also presents some of the frequent challenges in designing and implementing technical assistance, drawing from the existing literature and the authors’ experience in international development. The article summarises the latest thinking about delivering more effective development, including the adaptive management practices and the initiatives to strengthen evidence about what works. Finally, we complement this article with a follow-up open letter reflecting on the current policy options and opportunities for change.

## Disclaimer

The views expressed in this article are those of the author(s). Publication in Gates Open Research does not imply endorsement by the Gates Foundation.

## Introduction

Technical assistance is generally referred to as non-financial support, usually knowledge-based, contracted by and/or provided to governments by local or international experts to support policymaking and/or strengthen state capability.

The World Bank defines technical assistance as 'the transfer or adaptation of ideas, knowledge, practices, technologies, or skills to foster economic development for policy development, institutional development, capacity building, and project or programme support' ( World Bank, 1997). OECD DAC refers to technical assistance as technical cooperation provided in the form of: (i) grants to nationals of aid recipient countries receiving education or training at home or abroad, and (ii) payments to consultants, advisers and similar personnel as well as teachers and administrators serving in recipient countries, including the cost of associated equipment ( OECD DAC).

Technical assistance includes various practices, such as hands-on support, training, peer to peer learning, coaching, facilitation, embedding externals in the government, or South-South cooperation. In our complimentary paper on technical assistance ( Nastase *et al*., 2020), we refer to technical advisors providing support as DOERS, PARTNERS and/or FACILITATORS. We also refer to supply or demand-driven technical assistance. This article reviews the challenges of technical assistance provided to governments by external experts, using external funding.

The first seeds of international technical assistance were sown in the 1940s, with the establishment of the United Nations system and Harry Truman's ‘Point Four Program’ in 1949. Specifically, in the field of public health and academic capacity building, the Rockefeller Foundation established the paradigm of scientific neutrality in developing countries through the Foundation’s grants, projects, and fellows. For instance, its Mexico Hookworm eradication project, its viral research in India, the Caribbean, Brazil, and Egypt; and its Medical Sciences Division, which pioneered research in reproductive health etc.

In the 1950s, all through the 1970s, technical assistance was focused on building individual capacity in the government, primarily by strengthening technical knowledge and skills. This was channelled towards filling experience gaps in developing country governments and was achieved through the training of national staff and strengthening or restructuring government institutions. In general, this approach to technical assistance relied on technical experts from developed countries to work alongside and train recipient government staff. Over time, this led to core incumbent skills and functions that were supposed to be performed by the state officials being substituted by international technical advisers. It did not achieve the expected long-term goal of developing effective national institutions for the government ( Cox & Norrington-Davies, 2019).

From the 1980s onwards, technical assistance took a broader view of capacity, including some organisational elements. This was influenced by the introduction and development of the New Public Management approach, which transplanted business management practices to the public sector. Development organisations and governments have increasingly recognised, at least formally, that capacity development is about more than just building individual technical knowledge and skills. It was also an attempt to expand support to organisations to ensure sustainability of support beyond the individual bureaucrats as recipients of technical advice, knowledge or skills development programmes.

In the 1990s, research on strengthening state functions focused on the role of institutions, with research mainly on the roles of institutions in affecting economic development. The academic community has reached a consensus around the beginning of the 2000s that institutions matter, but the definitions of 'good institutions' are still under spirited debate ( Kiiza, 2006). The New Institutional Economists ( Hall & Jones, 1999; North, 1990) equated 'good institutions' with democracy, property rights, and the rule of law. The heterodox institutional analysts (Chang, 2002; Evans, 1995) saw the institutions as context-specific innovations that do not focus or start with Western or international best practices but rather with the country's realities and own values. In terms of capacity development, the novelty of the institutional level introduces the 'formal and informal rules' that complement the institutional and organisational level capacity. This framework is still used today by many development practitioners to understand capacity and how organisations develop, thus using technical assistance to enable reform ( UNDP, 2009).

The most recent shift in conceptualising technical assistance builds on complex systems theory. It postulates that ‘when smaller entities on their own jointly contribute to organised behaviours as a collective, [it results in] the whole being greater and more complex than the sum of the parts’ ( Paina & Peters, 2012). One of the main implications of the design and implementation of technical assistance programmes is that experimentation, flexibility, and learning become core programming elements. More flexible and adaptive models and research methods are required to accommodate these fundamental properties ( Smith & Hanson, 2011).

Considering each ecosystem in its uniqueness and making efforts to learning by experimenting, failing forward, adapting is thus more valuable than implementing blueprints and best practices. In practice, many programmes started to focus on asking process questions (‘how should we learn what works in the current situation?’), as well as trying to define best practice (‘what works?’) ( Husain, 2017). Another implication is that programmes built on the assumption of public institutions working within a complex system move away from rigorous outputs-based programme design to a commitment to results as part of adaptive log frames.

**Figure 1.**
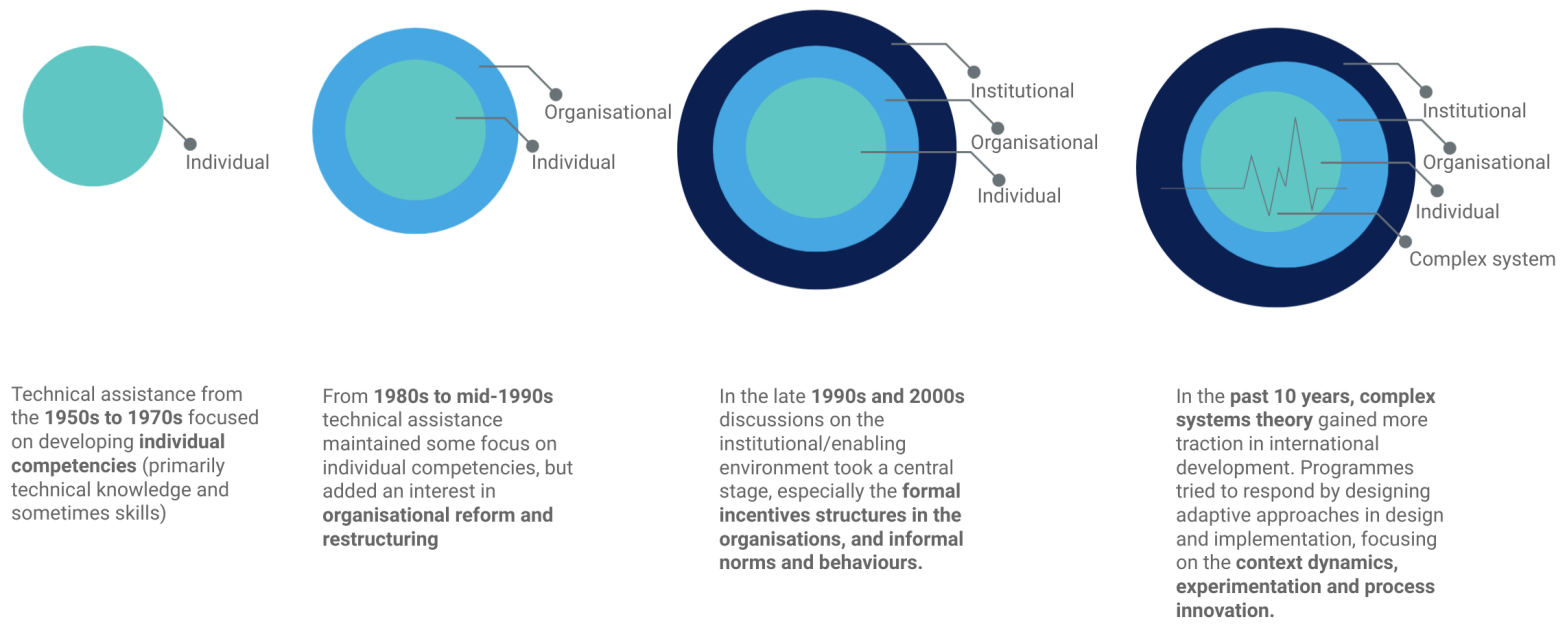
Evolution of technical assistance approaches linked to capacity development. *Each circle represents an area targeted for capacity development activities (individual, organisational and institutional capacity). In the last circle, the lifeline icon pulse shows that the system has a life of its own, and it is not only the sum of all the parts – the individual, organisational and institutional capacity*.

Figure 1 below summarises the evolution of the concept ‘capacity development’, starting from simply referring to building individual skills to working within a complex system.

## Application of technical assistance to health system strengthening

Over the years, technical assistance in public health focused on vertical disease programmes or health system strengthening. In the initial years, the focus was squarely on the former. Still, it was soon recognised that support to broader health system pillars is essential to ensure better delivery of vertical programmes ( Chee *et al*., 2013). This led to many donors pivoting towards health system strengthening projects, all with varying interpretations of what health system strengthening means and varying focus areas. Initial conceptualisations of a health system used a 'building blocks' approach – dividing a health system into six parts: health workforce, health information systems, supplies and infrastructure, finance, governance and leadership, and service delivery. Health system strengthening programmes have often focused on adding up interventions that address these individual building blocks.

In recent years, this mechanistic, linear view of a health system has been challenged ( Chee *et al*., 2013). It is gradually replaced by health systems seen as 'complex adaptive systems' or 'systems of systems', given the number of parts of a health system, all of which interact in complex ways. A field of applied research focusing on these issues – known as Health Services Research and Policy – has become increasingly prominent. At the heart of this paradigm change is the recognition that whilst the building blocks – which are seen as the 'hardware' of a system – are of course important, so are the people in the system – the 'software' ( Sheikh *et al*., 2011). Health systems strengthening should be approached through the dynamic interaction between the hardware and the software. For instance, the availability of resources affects providers’ motivations, or the incentives and norms affect the effectiveness of supplies and infrastructure. The extent to which hardware components translate into effective, quality service delivery depends on the behaviours and interactions of the people in a system. The behaviours are governed by 'tangible software' (their capacities and the formal processes that a system mandates) and 'intangible software' (the norms, values, incentives, relationships, and culture that influence behaviour in practice).

The implication of these conceptual advances in the health systems literature is that strengthening a health system can no longer be viewed as a technical, linear problem that can be solved through rational planning. Desired outcomes (whether resilience, performance, responsiveness, or respectfulness) are not things that can be simply achieved through strengthening inputs, particularly individual blocks. Instead, there is a need to create an enabling environment so that these outcomes emerge from the dynamic interactions between hardware, software, and different levels of the health system.

The health systems literature has made significant conceptual and empirical progress on identifying the enabling environment components required to achieve improved outcomes – particularly the importance of trust, pro-social values, teamwork, and distributed leadership. These tend to be shared across different types of desired results studied.

However, there has been significantly less progress in identifying interventions that can successfully bring about this enabling environment. Some of the interventions identified that have an emergent evidence base include training and coaching on supportive supervision, coaching, and mentoring on transformational leadership, the creation of peer networks, and social accountability. However, these only have limited traction in large health system strengthening projects – partially because they challenge the predominant approaches used in sizeable technical assistance programmes. There is no conclusive evidence on whether externally funded and provided technical assistance programmes are even the suitable vehicles for delivering systemic change in the 'software' of health systems despite having successfully diagnosed a problem.

## The principles of implementation of technical assistance

In 2005, the international community came together and agreed on principles that would guide effective aid. These referred to country ownership, alignment, harmonisation, managing for results, and mutual accountability. In 2008, an alliance including governments, civil society and international partners reinforced their commitment to three principles during the Accra meeting: ownership, inclusive partnerships, and delivering results. Later, the Global Partnership for Effective Development Cooperation was set up to support the achievement of Sustainable Development Goals. Their work is built on the principles agreed in 2011 in the Busan Partnership Agreement, and refer to country ownership, focus on results, inclusive partnerships, transparency, and accountability.

The authors concur that all these principles remain critical in providing effective aid and technical assistance. At the same time, our experience has shown us that the challenges lie in the implementation of the principles, including the day-to-day decisions and trade-offs, as well as the lack of repercussions for not following the principles. This article has modest objectives to summarise some of the frequently met challenges in implementation. However, these practical challenges need more rigorous research to be further documented and subsequently addressed.

## Key challenges for designing and delivering technical assistance – a practical account

**Country ownership** includes two essential conditions: countries setting their development priorities and development partners aligning their support accordingly while using country systems to channel their resources, including knowledge transfer (Global Partnership for Effective Development Cooperation). This principle would imply a nationally driven agenda and technical assistance as a policy option ( Nastase *et al*., 2020) for the country governments to deliver on their priorities.

Despite the almost unanimous agreement in the international development community that this is the essential principle in providing support to governments, many development projects still lack the needed ownership, and the results are less than ideal.

In practice, the reasons for the lack of ownership are both on the governments’ side and the donors’ side. Country ownership is difficult to define precisely and thus difficult to measure. First, at the national level, there is rarely ‘one country’ ‘one unifying voice’, ‘one vision’ for development, agreed collaboratively among relevant stakeholders and including clear development priorities ( OECD, 2012). Moreover, unstable political environments, with changing priorities, short-term visions, frequent government reshuffles, and weak bureaucracies do not create the enabling environment for ownership of a long-term development agenda ( Kiiza, 2006).

In practice, regular changes in political priorities, or the transfer of key personnel, is often a significant barrier to lasting change in government capability. Large-scale, long-term technical assistance programmes typically do not have the same leadership at the end of the programme or crucial decision-making points in the middle of the programme, as they started. Political and bureaucratic bipartisanship is often not a design feature of technical assistance programmes when they are launched. Frequent political, bureaucratic, and technocratic changes are also a defining characteristic of weak state capacity.

At the same time, donors also have their share of not being fully aligned to the ownership principle. The most documented reason is donors’ risk aversion, which is reflected in their reluctance to rely on the existing imperfect systems available at the national level. Their increased appetite for control is no safer, costs more and undermines long term development (OECD, 2011).

Attempts to transplant institutional models and best practice standards occur most often and have shown limited success in enabling governments to improve their functioning ( Andrews *et al*., 2012). While the assistance may help develop the technical systems and processes, it can be unsuccessful in changing the actual functions that an organisation performs, which is linked to individual behaviours ( Williamson, 2015). This challenge is not specific to lower-income countries. Still, it has more acute consequences when it repeatedly influences the projects, policies, and programmes, leading to capability traps – a cycle whereby governments are constantly adopting reforms, partly to ensure ongoing flows of external financing and legitimacy, without any tangible improvements. These capability traps emerge when there are efforts to reproduce templated solutions that are considered ‘best practice’ through predetermined linear processes or where there is an overbearing adherence to a rigid plan for implementation ( Andrews *et al*., 2012). For instance, there are indications that fragile and conflict-affected states are quicker to adopt results-based financing in health as compared to more stable countries, given their dependence on external funding but also weak governance arrangements ( Bertone *et al*., 2018).

Experience shows that poorly delivered technical assistance can displace and even erode national capacities ( Cox & Norrington-Davies, 2019). In addition, unintended capacity substitution can occur where poor working relationships exist between external experts and government staff. These relationships can be affected by disparities in salaries, equipment, and other softer elements, which lead to low morale within government counterparts ( Le *et al*., 2016).

The relationship between the government counterparts and external advisers depends, to a great extent, on the expectations on both sides. For instance, from our experience, these expectations are driven by path dependency, in the sense that government counterparts who have been receiving technical support for a long time in a particular shape, most frequently as capacity substitution, more prone to expect the same type of support with a prominent DOER role played by the external consultants. 

Technical assistance, by definition, refers to knowledge-based support. In this sense, the discussion about strengthening country ownership needs to reflect at least:

- What type of knowledge is considered useful, and how the different types of knowledge are valued?
- Who holds this knowledge, and what might be some existing preconceptions about who holds the knowledge?
- How knowledge is transferred, and how is it being used?

These concerns are converging in discussions about decolonising aid and those referring to more locally driven support. For instance, even if the public discourse is focused on integrating more local expertise, the tangible proof of following this principle is missing. More concretely, it would imply remunerating local expertise according to their experience and value added, compared to international expertise. This concern is exacerbated in some contexts, where ‘whiteness’ is the primary referent of power, prestige, and progress (Neajai Pailey, 2019). These stereotypes are only reinforced when the practice of inclusion and especially recognition does not follow the normative discourse ( Peace Direct, 2021).

In our experience, we have seen expectations from the government changing depending on the staffing of technical assistance programmes. For instance, junior consultants and external staff paid lower than government counterparts may end up doing capacity substitution work as they are perceived to be assistants for their government counterparts. On the opposite end, the senior consultants who may be compensated significantly above the government salaries may be accepted as partners or change facilitators. In some cases, working with the senior consultants also expects to intermediate the relationship with the superiors because communication within government systems is aligned with power and hierarchical rules.

**Focus on results** refers to achieving measurable results by using country-led results frameworks and monitoring and evaluation systems. **Transparency and accountability** refer to the arrangements through which countries and their development partners are accountable to each other and their respective constituencies. (Global Partnership for Effective Development Cooperation).

From our experience, the principle of managing for results is influenced in practice by the relationship between the donor and the implementer. The central tension characterising this relationship has to do with achieving results and managing risks. This is particularly obvious in the design stage, but especially during the implementation of a programme, depending on how this tension is handled.

One crucial element in qualifying this principle is to look at the accountability structures for reporting results. For bilateral donors, for instance, the primary accountability arrangement remains the domestic taxpayer to whom they need to prove value for money. For this, to have results under tighter control, donors come with their own programme-level results frameworks. These, sometimes, end up building counter bureaucracies that disrupt development projects ( Natsios, 2010) by focusing too much on predefined tasks. Predefined tasks are appropriate for simple problems, but they are not fit-for-purpose when dealing with complex problems in the public sector. Recent changes within the donor institutions that align development agenda more closely to domestic discourse ( Dercon & Dissanayake, 2020) could also affect the risk appetite and definition of results.

Other accountability structures worth mentioning are the ones within the donor organisations. What is valued within the organisation for individual career progression will inevitably affect how aid is delivered. Not much is being discussed or researched on how the internal performance frameworks shape the type of support provided. For instance, if 'business development' is much more valued in an individual performance review than facilitating development outcomes, this will inevitably create incentives for staff to expand their portfolio, irrespective of potential results.

Another relevant aspect to clarify is what results matter. The essence of aid is to support countries to become less reliant on external support and strengthen their capabilities. However, from our experience, many programmes are taking this aspect for granted as a by-product of any development programme. Instead, they focus on solving problems and achieving exclusively short and medium-term results, diverting the attention from building capability.

While ‘capacity development’ has become one of the most frequently used idioms in international development, its meaning has also been extracted. There is a generalised lack of clarity about how it can be achieved. For instance, a programme aiming to develop a parallel delivery system for health services and one that coaches managers to strengthen their leadership skills at work will have the same label of building capacity. More accurately, though, the first one is capacity substitution and should be recognised as such with its advantage of getting quick results and its limitation of potentially creating dependency on the external advisor instead of building in-house capability. The second one is building capacity because managers are supported to do their own job in their own context. At different times, in some contexts, both types of support may be needed. ( Nastase *et al*., 2020)

At the same time, getting quick results to showcase and building capacity on the long-run may be antithetic in some situations. However, sustained capacity in incumbent systems is built when the system discovers its problems – both small and large, tests their solutions and sometimes fail– essentially transforming itself into a thinking, learning and hence, a responsive and resilient organisation. External technical assistance needs to act concurrently and patiently in breaking the inevitable falls and accelerate learning of essential lessons without prejudice or predisposition towards a templated ‘solution’.

**Inclusive partnerships** refer to building inclusive development partnerships that are recognising the different complementary roles of all the actors in society.

Evidence shows that the collaborative design of technical assistance improves outcomes (Spoth *et al*., 2007). Research on the politics of reform in international development also indicates that coalitions are key to development for the simple reason that leadership is a collective process requiring inclusive, powerful, and legitimate partners ( Developmental Leadership Program, 2018). The theory of social change also emphasises the inclusiveness aspect of coalitions, by using the concepts of weak and strong ties ( Granovetter, 1973). The insights from the theory and practice of social change are that networks or coalitions of strong ties (‘people or groups like us’) are weaker than networks or coalitions of ‘weak ties’ (‘people or groups unlike us’). This is justified by the simple fact that networks of strong ties limit the capacity for change as they quickly create a closed-in, limited circle of people and resources. Weak ties, by contrast, includes people who are different, but they bring along different types and more resources and ideas.

In the past decade, also building on the success of the programme Coalitions for Change ( Bazeley, 2018), development programmes have increased their focus on building on-the-ground coalitions, networks of champions etc. to ensure the sustainability of the programme and working within the ecosystem. Some of the current challenges refer to the inclusion of the different actors in the design, not just during the implementation phase, to create more ownership and focus on building sustainable processes along the way. Inflexible theories of change and action of technical assistance programmes hamper the holistic visioning of the pathway to long-term impact and goals. No sub-system (e.g. health, agriculture, sanitation etc.) operates in a silo of its own: they have interdependencies with other sub-systems. Not considering these interdependencies and potential extraneous changes in the environment means that while much technical assistance has robust log frames and hypotheses, they ultimately have weak narratives on systemic and long-term change pathways. Also, the theories of change need to be anchored in the organisational and institutional context, which may also refer to understanding different work streams, including other ongoing initiatives of international partners, national authorities, and non-state actors.

**Harmonisation** (2005 Paris Declaration) refers to donors coordinating, simplifying procedures, and sharing information to avoid duplication. The structural challenge to implementing this principle is the projectised nature of technical assistance, which created silos between donor organisations, and sometimes even within the same organisation. Overlapping objectives with not enough coordination can undermine the development efforts.

For instance, in global health, many actors may be genuinely committed to addressing the issue of coordination, they face a series of challenges, such as the proliferation of global health actors; problems of global leadership; divergent interests; accountability issues; problems of power relations ( Spicer *et al.*, 2020). This often leads to projects not being strategic, and limited coordination leads to overlaps between donors, and, in certain cases, contradictory or inconsistent reform advice ( Cox & Norrington-Davies, 2019). External technical assistance providers are bound by their contract and terms of reference with the funding institution and the host government. This formally shifts the onus for coordination between external parties primarily onto the host government, which has a weak capacity, to begin with, perpetuating a vicious cycle dominated by an inter-partner dynamic where host governments take a backseat. Government ownership of critical reforms suffers in the process.

Technical assistance is also projectised in nature due to the way public procurement rules are structured in government. Building genuine capacity through technical assistance can have ambiguous pathways of change that need to be built along the way. Most government procurement structures are not designed to support projects of this kind but instead focus on making procurement better defined, with clear targets and deliverables and a strong focus on value for money.

## Learning from international practice in providing technical assistance

An underlying assumption in traditional models of public sector reform is that development issues are technical problems and arise out of information asymmetry. Hence, given better information about government performance and citizens’ entitlements, actors will behave differently, and the supply of public goods will improve. With the limited success of large-scale external technical assistance, this assumption has been challenged, and the literature suggests that coordination and collective action challenges influence the behaviour of actors much more than information asymmetry does ( Booth, 2014).

The past ten years in international development have been characterised by an increased interest in defining more politically astute programmes to address complex problems. At the foundation of this movement is the practitioners’ understanding that the mixed results of development programmes, and particularly capacity development programmes, have less to do with a lack of knowledge or funding and more with the power structures that allow actors, groups, or collective movements to gain from existing movements and to resist change ( Leftwich & Sen, 2011). As a result, different approaches emerged, involving principles, methods, and tools to operationalise more adaptive, politically informed ways of thinking and delivering technical assistance. These approaches include the following.

• **Thinking and working politically** is based on three core principles: strong political analysis, insight and understanding; a detailed appreciation of and response to the local context; and flexibility and adaptability in programme design and implementation ( TWP CoP, 2013).

• **Development entrepreneurship** is a form of thinking and working politically that postulates that development entrepreneurs work within a broader coalition building process, using iterative learning by doing and making small bets to find ways to introduce reforms ( Faustino & Booth, 2014).

• **Problem-driven iterative adaptation** rests on four principles: local solutions to local problems; pushing problem-driven positive deviance; trying, learning, iterating, and adapting; and scaling up through diffusion ( Andrews *et al.*, 2015)

• **Doing development differently** involves five potential starting points: swimming against the tide; working in and with government; feedback loops and data; organisational change; and diffusion ( Wild & Andrews, 2015)

• **Adaptive management** is an intentional approach to making decisions and adjustments in response to new information and changes in context ( USAID, 2016).

• **Learning sites** refers to technical assistance between researchers and health managers may build resilience, and this is achieved through researchers becoming embedded within the social networks surrounding and supporting health systems. Three learning sites were embedded in two different national contexts: in Kilifi County in Kenya, and in two health districts located in different provinces of South Africa. ( Gilson *et al.*, 2017).

A few principles are shared across these initiatives:

**Locally driven technical assistance focused on developing local capacity**. There is general acceptance that the only way technical assistance can create sustainable capacity is through ensuring that local problems are owned by and solved by local actors. Local actors are much more likely to have the motivation, credibility, knowledge, and networks to mobilise support, leverage relationships, and identify opportunities in politically astute ways, as compared to their external counterparts ( Andrews *et al.*, 2012). Mobilising local support is not a straightforward task. Therefore, technical assistance providers need to ensure that the problems they address have a high level of salience to local actors.

Gilson’s research on learning sites concludes that health system strengthening must pay closer attention to the software of health systems. Building resilience is not merely about equipping the system with more hardware and technology; it is also about developing people’s agency. As the author states: ‘resilience specifically is nurtured by developing the internal organisational capacities needed to adjust to and learn from routine challenges and preserve or even improve health system functioning. The forms of health system strengthening must guard against undermining these capacities’ ( Gilson *et al.*, 2017). The learning site experience suggests that the long-term process of health system change requires being embedded in the context, understanding complex and long-term processes and challenges, and maintaining the balance between genuine strengthening and cosmetic support. Learning sites are based on collaboration between researchers and health managers: research is conducted with practitioners rather than about their practice. Research-practitioner partnerships are formed between groups rather than individuals; they are multi-layered, dynamic, and interdependent ( RESYST, 2016).

**Focus on addressing smaller problems.** Two aspects require further consideration: focusing on problems instead of solutions and focusing on small bets to learn from, iterate, and scale-up. Scholars have discussed the need to shift from solution-focused development programming to a problem-driven approach for a long time. However, the practice has been slow to follow through on this. As we speak, best practice solutions are implemented worldwide in response to COVID-19, with little recognition for the particularities of context and understanding of the enabling conditions required for success. The problem is aggravated when the solutions are large reform programmes that capture many public resources. By contrast, entrepreneurship thinking suggests that many small bets are better than one large bet. Small bets make failure less costly and thus increase the degree of learning and innovation, and eventually, the effectiveness of the solutions.

**Diffusion, through positive deviance.** Part of the theory of change for some of the aforementioned approaches – although sometimes not explicitly mentioned – is positive deviance. This rests on the premise that the contexts in which development practitioners act are complex. For any given problem, someone in the community will have already identified a solution ( Green, 2016). Learning from how the solutions were possible in the first place, and supporting their diffusion within the system, makes a difference in development programmes. This is also the theory of change for some ‘pockets of effectiveness literature’ ( Hickey, 2019), although there is little evidence on their spill-over effects concerning the improvement of public sector performance outside the remits of the organisation these islands of high capacity ( Roll, 2014). Organisations are much more likely to pursue gradual and incremental reform, by resolving specific problems, rather than reform by following a set blueprint.

**Change as an adaptive and iterative process**. At least two types of problems are to be solved with technical assistance: logistical, with clear, proven ways of working; and wicked, involving system-wide approaches ( Andrews *et al.*, 2015). For the second category, there is a growing recognition that reform pathways are not linear, or even technical, but deeply rooted in recipient countries’ political and social context. This has led to finding ways of working adaptively and flexibly. The core of these new methods of working is defining portfolio approaches to solve problems, and deliberate ways of testing, learning, and experimenting while supporting change ( Cox & Norrington-Davies, 2019). The central assumption is that complex change programmes cannot rely on defining the entire journey to reform upfront. Instead, they require a clear definition of the destination and flexibility about the entry points to get to that destination.

Adaptive management is an umbrella concept for the adaptive approach to programme design and implementation, focusing on learning and adapting to get things done. Its premise is that the change we are usually seeking in development is complicated and sometimes unpredictable, and in these conditions, the decision-making process cannot be linear. It must be iterative. For example, starting a journey with a GPS navigator that does not update itself based on the encountered road conditions will not take you to your destination.

An adaptive management framework allows the programme implementers to draw on the relevant type of technical assistance at critical points to push for change. In literature, this is usually referred to as having a portfolio approach. The government has a menu of options to choose from, and they may build towards the end goal by experimenting with what works. For example, many development projects work on a model whereby they use in-sourcing to advance on an essential task that they do not have the capacity for but need to be delivered quickly (the demand-driven capacity substitution). Then they use specific technical inputs to understand a public sector delivery problem (capacity supplementation), which will be solved by the inter-departmental working groups facilitated by an external adviser (capacity development).

**Technical assistance providers as facilitators rather than doers.** At the heart of the emerging lessons is a growing recognition that a mix of approaches is required to solve complex problems. At the same time, if the wicked problem refers to budling capacity, evidence on adult learnings teach us that people skill up and change behaviours only when empowered, when having the opportunity to apply the skills in their day-to-day jobs and when their context is conducive to a change in behaviours and practices.

Complementary to the thinking on how to do development differently, other initiatives focused on improving the evidence base about what works in international development, including:

- **The World Bank’s Global Delivery Initiative** is a platform that aims to bring together knowledge of what works, and practitioners, to strengthen policy and programme delivery
- **J-PAL** conducts randomised impact evaluations to answer critical questions in the fight against poverty.
- **3ie** is an online repository of rigorous evidence on what works in international development, including evaluations, synthesis of studies, systematic reviews across sectors.

In recent years, various tools have been adapted for a systematic accumulation of knowledge about WHAT is being delivered and HOW is being delivered. Efforts to improve the knowledge base about the results of adaptive programmes have increased. They have departed from purely case studies done by the implementing partner to more sophisticated monitoring and evaluation systems. At the same time, more dedication from development partners is needed to share knowledge from testing and recording the use of different tools and approaches ( Pasanen & Barnett, 2019)

## Closing remarks

In a subsequent article, the authors discuss the current policy options for governments in using technical assistance and the opportunities for change.

## Data availability

No data are associated with this article.
