# Supplementary material for: Technical assistance: a practical account of the challenges in design and implementation
Source: Gates Open Res. 2021 Nov 19;4:177. Originally published 2020 Dec 3. [Version 2] doi: 10.12688/gatesopenres.13205.2 (PMC8920999; doi:10.12688/gatesopenres.13205.2)
Supplement: Peer review history

# Peer review: Reviewer response for version 2

**DOI:** 10.21956/gatesopenres.14632.r31431

**Published:** 2022-03-14

**Authors:** Maia King, Sierd Hadley, Simon Gill

**Affiliations:** 1 Department of Political Economy, King’s College London, London, UK; 2 Overseas Development Institute, London, UK

This paper offers a useful summary of the dominant schools of thought on technical assistance and how they have changed over time. Joining the governance and health community perspectives is also an important contribution to the literature that we know. As such, we believe this paper should be approved.

 However, we would like to note that the nature of the paper necessarily overlooks some of the more nuanced aspects of capacity building in its different forms and locations.

 For example, while it’s reasonable to suggest that institutional reforms are complex adaptive problems and necessarily require adaptation, in general terms, in practice most reforms also incorporate aspects that are more logistical in nature, and may be more amenable to typical project-based approaches to technical assistance (as noted in the book Building State Capability).

 Moreover, the evidence that adaptive approaches consistently deliver better results in donor-funded projects for capacity building is thin, which may be a reflection of the type of project that is suited to adaptive programming, the capacity of international organisations to work differently, or the fact that institutional reforms and capacity building are likely to have high failure rates in general.

 More generally, the narrative in the paper necessarily skips over more successful examples of longer-term capacity building (e.g. post-independence Botswana) and the role of incentives and quality in technical assistance providers. Given the aim to discuss both governance and health sector capacity building, the paper could also have drawn more extensively from the literature on efforts to connect the two disciplines in practice – i.e. to make institutional reforms more relevant for service delivery results, and to ensure health capacity building also supports broader efforts to reform the state.

 These comments should not hold the paper back from being approved; rather they are an indication of why a thorough debate on the future of technical assistance and capacity building is warranted, and how general arguments might need to be nuanced.

Does the article adequately reference differing views and opinions?

Yes

Are all factual statements correct, and are statements and arguments made adequately supported by citations?

Partly

Is the Open Letter written in accessible language?

Yes

Where applicable, are recommendations and next steps explained clearly for others to follow?

Yes

Is the rationale for the Open Letter provided in sufficient detail?

Yes

Reviewer Expertise:

Researchers and practitioners on development practice.

We confirm that we have read this submission and believe that we have an appropriate level of expertise to confirm that it is of an acceptable scientific standard.

# Peer review: Reviewer response for version 1

**DOI:** 10.21956/gatesopenres.14418.r30095

**Published:** 2021-03-15

**Authors:** Maia King, Simon Gill, Sierd Hadley

**Affiliations:** 1 Department of Political Economy, King’s College London, London, UK; 2 Overseas Development Institute, London, UK

**Rationale for the paper:**

- This paper brings together in a concise and accessible way the history of discussions on technical assistance and capacity building more broadly. This provides a helpful framing for a range of issues on the topic of technical assistance. The comparison of the broader governance debates with the literature on health systems strengthening is also a very important contribution.

**Framing and definitions:**

- There are some aspects of the narrative and framing which could be clearer. The paper’s definition of technical assistance is “ *knowledge-based technical assistance contracted by or provided to government to shape effective and inclusive policies, strengthening policy delivery, and building government capability.” *This is very general, almost tautological, and does not mention the day-to-day practical reality of TA, e.g. training or the role of external consultants.
- The introduction also suggests that the paper will consider technical assistance as including government-contracted support that is not provided or funded by external actors. But in fact, the content of the paper is mostly relevant to cases when external actors do play a role, so this broad framing does not seem to add much. And does the paper’s definition of TA include peer learning in the OECD or other fora, or South-South Cooperation?

**Broader issues that could be addressed:**

- **The diversity of TA approaches** and what has been learned about how these work, for different kinds of challenges and contexts. Most of the paper is about wicked problems, though it also cites more linear approaches, but does not refer back to the Building State Capabilities chapter on this. Second, there could be some more analysis of the experience with transitioning from gap filling to capacity building. Positive long-term examples include Mauritius and Botswana.
- **Localising learning and building longer-term resilience.** The article is excellent in highlighting the need for much better local problem definition of the issues to be solved. Further discussion could be added on what practical steps by external actors could support this. For example, external providers could be asked to adhere to a stronger code of conduct to avoid inappropriate TA provision.
- **The practicalities of implementation.** While thinking has moved significantly there has been an absence of change in the areas of evaluation and procurement, which therefore inhibit both the design and delivery of more innovative solutions. The portfolio approach mentioned could be one way to get around this. It would also be useful to have some examples of good TA programming in the health sector.

**Referencing and citation:**

- It would be most helpful for the authors to add further clarification on which parts of the paper are drawn from their extensive professional experience and which are drawn from the literature.
- It would also be helpful for the authors to set out the specific contribution of the paper and to distinguish what is new and different compared to other papers, for example, Cox and Norrington Davies (2019).
- Further sources that the authors may also want to consider include:
  - A governance practitioners notebook (OECD 2015).
  - The seminal DFID publication which summarised thinking on TA: ‘Promoting Institutional and Organisational Development’ (2003).
  - Literature on capacity-building: Baser and Morgan (2008), Brinkerhoff and Morgan (2009), Hosono *et al.* (2011), Horton *et al. *(2010), Mallet *et al.* (2014), Morgan (2009).
  - Knack *et al. *(2020 ^1^) on ‘How Does the World Bank Influence the Development Policy Priorities of Low-Income and Lower-Middle-Income Countries?’
  - Solter and Solter (2013 ^2^) on ‘Providing technical assistance to ministries of health: lessons learned over 30 years’.

**Minor editing points:**

- The grammatical error in the phrase “ *technical assistance is a policy options".*
- P4: Mimicry is thought to be more prevalent in lower income country settings, but this is not really proven in any of the literature. For example, the UK is accused in a range of literature of implementing reforms that make little difference to overall organisational performance.
- P4-5: quite a few broad-brush statements without much indication of where these ideas came from: pressures on consultants, time to have impact, risks, frequency of changes to goals, etc. Are these the views of the authors or are they reflected in the broader literature surveyed?
- P6: the approaches listed arguably reflect two different camps: (a) the adaptive development agendas; (b) for want of a better term, the evidence agenda. This suggests either the Global Delivery Initiative needs to be described as an adaptive approach or listed along with other JPAL, IGC, ISS, etc.?
- P7: this sentence was not clear: “This is exacerbated in places where the legislature (i.e. politicians) cannot or does not fully rely on the executive to implement most of its policies”.

**An addendum: The impact of a changed world**

 The impact of COVID-19 will have a significant impact both on how TA is delivered (particularly if it is based on international exchange) and also on the type of support that will be needed to equip organisations for the changed and changing realities around them.

 There is a danger that ‘capacity development’ is seen as a static concept based on the existing status quo and therefore is designed to address issues like improving service delivery across a Ministry, increasing efficiency or capacity. However given the uncertain, dynamic nature of the world we live in (now exacerbated by COVID-19), we need to help institutions/organisations and the individuals who are part of them to adapt to new and changing situations. Given COVID-19 has radically changed the world, we need to find a way to promote resilience and adaptive capability as well as developing capacity.

 Therefore, as well as changing the type of support that needs to be provided, the impact of the pandemic will accelerate the need to equip and support local actors. They increasingly need to be the ones to develop and use adaptive, politically-smart, incremental etc. practices if these are the most helpful for policy design and implementation. The lens of donor approaches has so far often been on how to make donor programmes more effective. But there will need to be a change of emphasis towards methods that genuinely support locally driven change, and ensure it is both embedded and creates a drive for ongoing reflection and adaptation. This will need to be built on the abilities of local actors to analyse local problems and to develop responses built on their own strengths and abilities.

Does the article adequately reference differing views and opinions?

Yes

Are all factual statements correct, and are statements and arguments made adequately supported by citations?

Partly

Is the Open Letter written in accessible language?

Yes

Where applicable, are recommendations and next steps explained clearly for others to follow?

Yes

Is the rationale for the Open Letter provided in sufficient detail?

Yes

Reviewer Expertise:

Researchers and practitioners on development practice.

We confirm that we have read this submission and believe that we have an appropriate level of expertise to confirm that it is of an acceptable scientific standard, however we have significant reservations, as outlined above.

## References

[1] : How Does the World Bank Influence the Development Policy Priorities of Low-Income and Lower-Middle-Income Countries. *Policy Research Working Paper* .2020; Reference source

[2] : Providing technical assistance to ministries of health: lessons learned over 30 years. *Glob Health Sci Pract* .2013;1(3) : 10.9745/GHSP-D-13-00121 302-7 10.9745/GHSP-D-13-00121 25276545PMC4168592

# Response

**Published:** 2021-09-07

**Authors:** Alexandra Nastase

**Affiliations:** Oxford Policy Management, UK

Thank you for taking the time to review our article and for all your thoughtful comments. We have now revised the document in line with the suggestions received.

 Please find below our responses:

**Rationale for the paper: **We agree. Our intention was to provide a quick summary of the existing relevant literature on the topic. We chose this format of open letter as it allowed us to share our practical experience as well, which we considered relevant to cover practical aspects of designing and implementing technical assistance programmes. 

**Framing and definitions**:

- We agree with your comment on the definition and we updated it in the current text. Also we linked this open letter to a complementary paper we have written about the implementation practicalities  and the opportunities for change.
- We clarified the type of TA we will be referring to
- We included references to methods of delivery of TA, including training, South South cooperation etc.

**Broader issues that could be addressed:**

- We agree that some of the references to types of problems were confusing. We tried to clarify this in the revised version. We also tried to give more nuance to the type of support needed – particularly in the complementary open letter on TA models
- We added one small section on transition, with reference to strengthening institutions in Botswana and Mauritius in the complementary paper that is more forward looking.
- Agree on the suggestions on further localizing aid. We have explored this topic further together with recommendations such as a code of conduct, more appropriate remuneration for local staff etc. (these are included both in this article and the complementary open letter)
- Agree on the practicalities of implementation. We added some ideas in this complementary article:  https://gatesopenresearch.org/articles/4-180/v1

**Referencing and citation:**

- We improved the referencing and citation, hopefully satisfactorily
- This open letter tried to summarise some of the challenges to set the scene for the open letter referring to policy options and opportunities for change. We have built on the existing literature, but referred to other elements such as the health sector TA, the references to system thinking, as well as referring to practical challenges within the 2005 Paris Declaration.
- We have included all the suggested additional bibliography

**Editing:**

- We thank you for all the editing suggestions. We clarified the ideas in the current revised text and we modified where necessary.

**The addendum:**

- We agree a comment was necessary on the potential changes arising from the COVID-19 pandemic. We considered this section to be appropriate for the complementary present an forward facing open letter.

We hope this responds satisfactorily to your comments and suggestions.

# Peer review: Reviewer response for version 1

**DOI:** 10.21956/gatesopenres.14418.r30291

**Published:** 2021-02-26

**Authors:** Brendan S. Whitty

**Affiliations:** 1 School of International Development, University of East Anglia, Norwich, UK

This paper provides a useful overview of the development of technical assistance programming and the policy ideas that have dominated it, from the 1970s to the present day. The main focus of the paper is an account of the challenges faced by technical assistance and the literature on ‘isomorphic mimicry’ and the importance of flexible and adaptive practice that is primarily facilitative in nature.

 While it does not seek to address the challenges mounted by critical accounts of the challenges in ‘capacity building’ under the conditions and power relations frequently present in technical assistance programming (and the difficulties in facilitating change under conditions of heterogenous interests and institutions), the key concepts and issues involved in the policy literature on this kind of technical assistance are capably reviewed, and the article identifies the key learning well. It is a perfectly serviceable review of the current state of the art on PDIA and complexity-driven adaptive programming in the field of technical assistance, without adding much and without addressing the critical literature (which it makes no claims to do).

Does the article adequately reference differing views and opinions?

Partly

Are all factual statements correct, and are statements and arguments made adequately supported by citations?

Yes

Is the Open Letter written in accessible language?

Yes

Where applicable, are recommendations and next steps explained clearly for others to follow?

Yes

Is the rationale for the Open Letter provided in sufficient detail?

Yes

Reviewer Expertise:

International development practice

I confirm that I have read this submission and believe that I have an appropriate level of expertise to confirm that it is of an acceptable scientific standard.

# Response

**Published:** 2021-09-07

**Authors:** Alexandra Nastase

**Affiliations:** Oxford Policy Management, UK

Thank you for taking the time to review our article. We have now revised the document in line with the suggestions received.

 Please find below our responses:

- We tried to incorporate more comments on the existing power structures that make change difficult, building on existing literature and our experiences.
- We have also revised a complementary open letter that provides a framework to categorize the existing types of technical assistance and potential opportunities for change.
- This article can be accessed here: https://gatesopenresearch.org/articles/4-180/v1

We hope this responds satisfactorily.
